# COVID-19 Outbreak Associated with Air Conditioning in Restaurant, Guangzhou, China, 2020

**DOI:** 10.3201/eid2609.201749

**Published:** 2020-09

**Authors:** Francis W. Moses, Ricardo Gonzalez-Rothi, Gene Schmidt

**Affiliations:** Moses Engineering, Gainesville, Florida, USA (F.W. Moses);; Florida State University, Tallahassee, Florida, USA (R. Gonzalez-Rothi);; Schmidt Consulting Group, Pensacola, Florida, USA (G. Schmidt)

**Keywords:** airborne viral spread, indoor air conditioning, respiratory infections, severe acute respiratory syndrome coronavirus 2, SARS-CoV-2, COVID-19, 2019 novel coronavirus disease, coronavirus disease, zoonoses, viruses, coronavirus, air conditioning, airborne transmission

**To the Editor:** Lu et al. (*1*) describe the indoor airborne spread of COVID-19 (coronavirus disease) facilitated by a type of standard, wall-mounted, ductless air conditioner (AC) used in most countries. These units are low-cost in comparison to ducted AC units, which can cost 3 times as much to purchase, install, and operate. Ductless units cool and dehumidify indoor air but have little ability to filter or remove airborne contaminants.

A wall-mounted ductless system blows air directly onto those closest to it, potentially disseminating infectious droplets or aerosols along the airflow. Lu et al. use arrows to point out the airflows emanating from and returning to the AC unit, delineating a possible trajectory of putative airborne droplets. This trajectory coincides with the seating distribution of other persons at the restaurant who later became ill (*1*). We agree that the AC probably contributed to the upstream and downstream airborne spread of the virus.

The type of AC system required to mitigate airborne transmission is neither affordable nor architecturally feasible for many buildings or regions. To prevent the spread of coronavirus disease in indoor spaces, we need work-around solutions in addition to distancing and fresh air exchange. Viable, low-cost possibilities might include operating AC on low fan settings and installing units near the ceiling, which would channel airflow towards the ceiling instead of directly onto patrons. Other methods might include installing high-efficiency particulate air filters, ultraviolet germicidal irradiation (which can disinfect some airborne coronaviruses such as mouse hepatitis virus and Middle Eastern respiratory syndrome coronavirus) (*2*), or a combination of these methods.
